# Unproven medical devices and cancer therapy: big claims but no evidence

**DOI:** 10.2349/biij.4.3.e25

**Published:** 2008-07-01

**Authors:** MP Mac Manus

**Affiliations:** Department of Radiation Oncology, Peter MacCallum Cancer Centre, Melbourne, Australia

The scientific method is an affront to the advocates of the irrational, magical or pseudo-scientific belief systems that characterise “alternative” or “complementary” therapies such as homeopathy, craniosacral therapy and ear candling, to name but a few of the less credible varieties. Practitioners of these so-called therapies are unable to provide any verifiable explanations as to how these might work but they nevertheless claim that they can restore or improve health with their natural and gentle healing methods. Some practitioners claim that they can cure or control cancer and they are often sought out by desperate patients or their families. Unfortunately, no consistent bodies of high-quality evidence exist to show that their therapies or methods work as claimed or are better than the placebo effect. In fact, the claimed benefits of alternative medicines are most often described in glowing testimonials and advertisements rather than in scientific papers. Despite the fact that many alternative or complementary therapies are claimed to embody ancient wisdom, there is a surprising lack of well-conducted research, carried out to see if these therapies actually work. When rigorous testing is done, the benefits of alternative therapies are swiftly reduced to the placebo effect. If a treatment has a sound scientific basis and is proven to work reproducibly, then it is not alternative medicine – it is just medicine.

Many herbal medicines are effective because they contain pharmacologically active substances derived from plants. For example, St John’s Wort has been shown to be helpful for some patients with depression. Unlike modern pharmaceuticals, herbal medicines vary in their strength from batch to batch because they may contain unpredictable amounts of the active substance that produces the therapeutic effect. Herbal medicines may also contain other substances that are toxic or antagonistic to the effects of the therapeutic components of the plant. Many modern medicines were first isolated from plants used in herbal medicine, but are now produced by the pharmaceutical industry in pure form, in metered doses and uncontaminated by other molecules. However, in societies where modern medicine is unavailable or available only to the rich, a consultation with a herbalist or other non-medical practitioner may be the only source of healthcare.

It is easy to understand why alternative and complementary therapies flourish when no other help can be found. However, such therapies are also popular in prosperous countries with relatively well-educated populations. When conventional cancer treatments have failed to cure, patients will try any method that seems plausible and fits in with their world view. Despite the lack of scientific evidence, many people find the philosophies and magical belief systems underlying many alternative medicines to be very attractive. Practitioners offer hope when all seems lost. Modern medicine can seem impersonal, rushed and blind to the needs of the individual. Alternative practitioners can more offer time and empathy than is usually available from conventional practitioners. Some parts of society harbour a significant amount of hostility to the “pharmaceutical industry” and the “medical establishment”. There is suspicion that the benefits of natural healing systems are being concealed to keep profits up. New Age beliefs and dissatisfaction with conventional medicine can make patients easy prey for unscrupulous providers of dubious alternative therapies. Patients may receive advice that is frankly dangerous. Many homeopathists in the UK are opponents of vaccination and some have even promoted homeopathic treatment (sugar pills) instead of antimalarial prophylaxis; advice that will kill people travelling unprotected to endemic areas.

There is no doubt that many alternative practitioners actually believe that their therapies work. In recent years there have been efforts of varying sincerity to test some of these therapies. Unfortunately, the bulk of scientific studies of alternative or complementary medicines have been poorly-designed and poorly-controlled, and are published in journals with less-than-rigorous peer review. As the more prominent alternative therapies such as acupuncture and homeopathy are tested in ever more rigorous and well-designed trials, their apparent benefits progressively disappear. A prime example of this occurred in a recent study that compared acupuncture with sham acupuncture and standard therapy in the management of 1162 patients with back pain [1]. Interestingly, both groups of patients treated with either “real acupuncture” (using needles placed on correct traditional Chinese acupuncture points) or sham acupuncture (using superficial needling at non-acupuncture points) did about twice as well as patients given the standard therapy of drugs, physical therapy and exercise. So, does acupuncture work? Unfortunately not in this study! The results for “real” and “sham” acupuncture were not significantly different. Needle placement on acupuncture points was not necessary. This study suggests that a consultation that involves placing needles under the skin and a belief that this is part of an ancient system of medicine is enough to invoke a very powerful placebo effect in very many people. Five large meta-analyses have investigated the evidence for homeopathy. All have had the same result: after excluding methodologically inadequate trials and accounting for publication bias, homoeopathy produced no statistically significant benefits over placebo [2].

Alternative and complementary approaches to treatment may involve the use of unproven “medical devices”; pieces of “technology” that are claimed to have diagnostic or therapeutic properties. One of the simplest and most ridiculous is the so-called traditional Hopi Indian ear candling. This is a method that involves placing a lighted hollow candle in the ear to remove wax. Some practitioners have also claimed that it can “draw toxins” from the body. Vanessa Charles, public relations officer for the Hopi Tribal Council, has stated that ear candling "is not and has never been a practice conducted by the Hopi tribe or the Hopi people”. The method is not only of doubtful provenance, but is ineffective and has led to injuries. Professor of Complementary Medicine at Universities of Exeter and Plymouth, Edzard Ernst has published critically of ear candles: “There are no data to suggest that it is effective for any condition. Furthermore, ear candles have been associated with ear injuries. The inescapable conclusion is that ear candles do more harm than good. Their use should be discouraged” [3].

Why am I discussing alternative medicines at such length in an article about unproven medical devices used in cancer therapies? Unfortunately, too many patients are being offered useless treatments for cancer that use unproven medical devices, or misuse real medical devices. Claims made for these medical devices are the same sorts of claims made for older and better established forms of alternative medicine. The same sorts of justifications and similar poor-quality evidence are used to impress patients (or victims, as I prefer to call them). The practitioners often have better pseudoscientific jargon than most alternative practitioners, and they also have an impressive-looking machine of some sort. In some cases, the practitioners even have medical degrees. Some of these therapies are clear cases of medical fraud, others are just plain old-fashioned quackery, and perhaps others represent a sincere failure of judgement or even a delusion on behalf of the therapist. The claims made for these devices are often false, distorted, or at best, unsupported by evidence. Many victims suffer financial losses, bad health outcomes or both. The most tragic cases are those in which patients with potentially curable cancers forsake proven therapies for quackery.

The same types of advertising are used as for other alternative therapies, especially on the Internet where regulation ranges from lax to non-existent. Advertising emphasises anecdotes and testimonials but never quotes the most relevant type of research: the controlled clinical trial. However, unlike many other types of alternative medicine, the claims for these medical devices are dressed up with plausible-sounding bits of scientific jargon. The therapist may say that this treatment will “help the immune system fight cancer” or that it will “starve the cancer of the glucose it needs to survive”. The therapist may use a device that will “scan your body” or “analyse” your blood or a hair sample and detect critical nutritional deficiencies or imbalances that you need to correct to survive your cancer. The therapist will claim to have special knowledge that is not accepted by the established medical profession. Conventional cancer medicine is determined to “slash, burn and poison cancers” they say, instead of adopting a gentle and more reliable method, with the unproven medical device at its centre. The patients may be assured that conventional medical specialists are aware of the fantastic scientific advances enshrined in the unproven medical advice but are determined not to accept this knowledge for fear of losing their livelihoods. On the other hand, the medical world may be too stupid to appreciate the genius of the therapist. The patient may be assured that the huge international efforts involved in the study of difficult sciences such as molecular biology, immunology pharmacology, radiation biology, etc, is a shameful waste (perhaps even a deliberate waste) when the problem of cancer can be reduced to a few simple concepts that the average person can grasp. Ideas such as “working with your body rather than against it to fight the cancer”, “sorting out your lifestyle” and “taking responsibility for your own illness” make the patient think of the therapist as a concerned individual offering a real alternative to the nasty treatments provided by conventional cancer specialists. A holistic approach and “empowerment of the patient” should, of course, be part of good quality cancer care from any source. The difference is that truth should be the basis of any treatment approach, and the truth is in short supply when these unproven medical devices are promoted.

For these devices or methods, no proper clinical trials are done or discussed. No phase I trials to assess toxicity, no phase II trials to assess efficacy and no phase III trials to compare with standard therapies. There is no ethics committee approval and patients are not asked to sign a consent form stating that they are enrolled in a trial of an investigational therapy. However, they may be asked to sign a waiver to “cover” the person treating them, acknowledging that the therapy is not accepted by the medical establishment. This, they are assured, does not mean that the treatment does not work, “it is just a legal requirement to satisfy the regulators”. The practitioner may adopt the wry smile of an embattled innovator struggling with the uncaring forces of government regulation. The therapist may claim to be conducting research, but there is no ethics approval and the results of well-conducted clinical trials are not published in respected journals.

Then there is the money. No matter how simple the treatment seems, it will be expensive. It may seem tailored to the amount that the person seems likely to be able to afford. Special discounts may be offered to those with less money, or a cheaper “but just as effective” form of the therapy may unexpectedly become available for those with financial problems. Often treatment with the unproven medical device is just one of a menu of treatments available at an alternative cancer treatment centre. One may also find homeopathy, iridology, naturopathy, orthomolecular medicine and other mutually contradictory members of the complementary and alternative medicine family available.

The types of unproven therapies used to treat cancer patients with “medical devices” vary considerably. Usually they are completely without evidence other than anecdotes. The types of devices include so-called “energy machines” that can supposedly cure cancer and eliminate AIDS by transmitting electromagnetic waves through the body. The “Rife” machine has been claimed to be effective against cancer by causing "differentiation" of cancer cells into normal cells by eliminating microorganisms that caused the cancer. There is no evidence to support this therapy but it is widely available. Some therapists use a box called a “magnetic pulser” and claim that it can shrink cancers. In the UK, a practitioner was found guilty under the trades descriptions act after using an “IFAS High Frequency Therapy” machine to treat cancer. Other methods are more subtle and represent a misuse of existing and proven therapeutic technologies. For example the practitioner may offer whole body hyperthermia to a patient, using a microwave machine that is unable to induce whole body hyperthermia to a temperature that is effective for killing cancer cells. The practitioner may offer local hyperthermia with a machine that is incapable of heating the patient’s deep-seated tumour to an effective temperature. The patient may be offered photodynamic therapy for a deep-seated tumour, when the therapist knows very well that the laser beam used in the therapy is unable to penetrate deeply enough into tissue to treat the tumour effectively.

Regulation in this area is lax in many countries. Governments do not like to be seen to be limiting patient choice. There are, however, occasional comprehensive reviews of unproven therapies by scientific bodies. In 2005 the Australian National Medical and Health Research Council reviewed a form of “microwave therapy” delivered with so-called “glucose blockers” and found that there was no high-quality published scientific evidence which showed superior benefits in terms of therapeutic effectiveness for the treatment of cancer with microwave (or UHF) cancer therapy when combined with radiotherapy or “glucose blockers” [4]. Despite these findings, the therapy remains available.

This is a tragic problem and I must confess that sometimes it makes my blood boil. I have seen patients who have wasted large sums of money that they or their families could ill-afford. Some patients have listened to the advice of quacks and refused conventional treatment for cancer at a time when they could have been cured. Instead they have accepted useless treatment in which a “medical” device was used or misused. Others have suffered needlessly with severe symptoms that could have been readily relieved with conventional anti-cancer therapies. These devices are unfortunately just part of the spectrum of alternative and complementary medicine, dressed up in a coat of pseudoscience, giving the impression to a person with a limited understanding of science that they represent an exciting advance being held back by the corrupt medical establishment. Some of the practitioners of this form of fraud are clearly heartless predators. Members of the medical profession who deliberately practise fraud should be de-registered. Those who recklessly endanger life and cause suffering to vulnerable patients and their families for profit deserve even more serious penalties. Those of us who use evidence-based medicine in the treatment of cancer need to be alert to the size of this problem. We need to offer patients good advice and we should warn them of the dangers that they may face when, all too understandably, they seek opinions from purveyors of unproven therapies. We should empower them with information so that when confronted by an alternative practitioner they will ask, “show me the evidence that what you say is true”.
